# Vision impairment and cognitive function in Chinese older adults with diabetes: multiple mediating effects of social participation and perceived control

**DOI:** 10.3389/fpubh.2026.1952426

**Published:** 2026-09-09

**Authors:** Ying Yin, Tingting Wang, Ting Xu, Yulu Liu

**Affiliations:** 1 Emergency Department, The First Hospital of Hunan University of Chinese Medicine, Changsha, Hunan, China; 2 Department of Nursing, The First Hospital of Hunan University of Chinese Medicine, Changsha, Hunan, China; 3 Department of Urology, Yueyang People’s Hospital, Yueyang, Hunan, China

**Keywords:** cognitive function, diabetes, multiple mediation effect, perceived control, social participation, vision impairment

## Abstract

**Background:**

Vision impairment and cognitive decline are common concerns among older adults with diabetes. Although visual difficulties have been associated with poorer cognitive function, the social and psychological pathways underlying this association remain insufficiently understood. This study aimed to examine the independent and serial indirect roles of social participation and perceived control in the association between vision impairment and cognitive function among Chinese older adults with diabetes.

**Methods:**

Data were obtained from the 2018 wave of the Chinese Longitudinal Healthy Longevity Survey. A total of 1,072 Chinese adults aged 65 years or older with diabetes were included. Descriptive statistics were used to summarize participants’ sociodemographic and health-related characteristics. Spearman’s rank correlation analysis was conducted to examine the associations among vision impairment, social participation, perceived control, and cognitive function. A serial multiple mediation analysis was performed using Model 6 of the SPSS PROCESS macro version 4.0. Age, years of education, sex, place of residence, self-reported quality of life, and self-rated health were included as covariates. Indirect effects were estimated using 5,000 bootstrap samples and bias-corrected 95% confidence intervals. To assess the robustness of the mediation results to different covariate-adjustment strategies, sensitivity analyses were additionally performed using an unadjusted model without covariates and a fully adjusted model including all candidate covariates.

**Results:**

Vision impairment was significantly associated with poorer social participation, poorer perceived control, and lower cognitive function, and all study variables were significantly correlated with one another (*p* < 0.01). After adjustment for the mediators and covariates, greater vision impairment remained significantly associated with lower cognitive function (direct effect = −0.8085, SE = 0.1971, 95% CI: −1.1952 to −0.4218). The total indirect effect through social participation and perceived control was statistically significant (effect = −0.0783, SE = 0.0317, 95% CI: −0.1466 to −0.0215). The indirect pathway through social participation alone was significant (effect = −0.0484, SE = 0.0226, 95% CI: −0.0965 to −0.0089), whereas the indirect pathway through perceived control alone was not statistically significant (effect = −0.0260, SE = 0.0235, 95% CI: −0.0830 to 0.0090). The serial indirect pathway through social participation followed by perceived control was statistically significant (effect = −0.0038, SE = 0.0026, 95% CI: −0.0102 to −0.0002).

**Conclusion:**

Greater vision impairment was associated with poorer cognitive function among Chinese older adults with diabetes. Social participation showed a significant independent indirect association, while perceived control alone did not. However, social participation and perceived control jointly formed a significant serial indirect pathway. These findings suggest that social participation and perceived control may be relevant to understanding the association between vision impairment and cognitive function. Given the cross-sectional design, the temporal sequence and causal direction of these relationships require confirmation in longitudinal studies.

## Introduction

Against the backdrop of continued improvements in life expectancy and persistently low fertility, China’s population is aging rapidly, with an increasingly pronounced shift toward older age groups within the older population (1). Population aging is reflected not only in the expanding number of older adults but also in the growing burden of age-related functional decline, multimorbidity, and cognitive impairment, thereby placing increasing demands on healthcare and long-term care services (2). Cognitive function is fundamental to older adults’ ability to maintain independent living, manage chronic conditions, and participate in daily and social activities (3). Lower cognitive function is associated with limitations in activities of daily living, poorer quality of life, greater use of unplanned healthcare services, and a higher risk of all-cause mortality (4, 5). Therefore, identifying potentially modifiable factors associated with cognitive health is important for promoting healthy aging.

### Vision impairment and cognitive function among older adults with diabetes

Diabetes imposes a particularly substantial disease burden on older adults in China. A recent study estimated that approximately 233 million people in China were living with diabetes in 2023, with an age-standardized prevalence of 13.7%. The prevalence increased with age and exceeded 30% among older age groups (6). These findings indicate that diabetes has become a major chronic condition affecting the health of older adults in China. Visual impairment is a common complication and coexisting health problem among people with diabetes. Diabetes can affect the retina, resulting in diabetic retinopathy and diabetic macular edema, and is also associated with the earlier or more frequent occurrence of cataracts, glaucoma, and other ocular conditions that may compromise visual function (7). In addition to restricting daily activities, visual difficulties have been associated with poorer cognitive performance and a greater risk of cognitive decline among older adults (8, 9). Particularly in individuals with diabetes, visual and cognitive impairments may partly share underlying vascular, metabolic, and neurodegenerative processes (10). However, biological mechanisms alone may not fully account for the association between visual impairment and cognitive function. It is therefore necessary to further examine the potential social and psychological factors involved in this association among older adults with diabetes.

### Vision impairment, cognitive function, perceived control, and social participation

Social participation may represent an important social and behavioral factor for understanding the association between visual impairment and cognitive function. It generally refers to the extent to which individuals interact with others in social settings and engage in recreational activities, community organizations, volunteer work, or other group-based activities (11). Visual function is an important prerequisite for older adults to obtain information from their surroundings, maintain independent activity, and participate in diverse social contexts (12). Previous studies have found that older adults with visual impairment generally report lower levels of social participation than those without visual impairment, and that the range and frequency of their social activities may also be restricted to varying degrees (13, 14). Among older adults with diabetes, visual difficulties may coexist and interact with physical functional limitations, multimorbidity, and complex disease-management demands, thereby creating additional barriers to maintaining everyday social relationships and participating in community activities (15, 16). Social participation is also closely related to cognitive health in later life. Existing evidence indicates that higher levels of social participation are associated with better cognitive performance and a lower risk of cognitive decline (17). Continued engagement in social activities may help older adults remain connected to their external environment and gain access to diverse behavioral and psychological resources (18). Social participation may therefore be a potentially modifiable social and behavioral factor in the association between visual impairment and cognitive function.

Perceived control may represent another important psychological factor related to cognitive health. It refers to an individual’s subjective belief that they can influence life events and their outcomes, respond to environmental demands, and manage everyday affairs (19). In later life, chronic illness, declining functional capacity, and increasing reliance on others for care may limit an individual’s ability to independently manage daily tasks and influence the organization of everyday life, thereby challenging autonomy and perceived control. Maintaining a certain degree of perceived control may therefore constitute an important psychological resource for adapting to changes in health and living circumstances (20, 21). Limitations in independent mobility, difficulties managing daily affairs, and an increased need for external assistance associated with visual impairment may be related to a weaker sense of independence and lower perceived control among older adults (22). Previous studies have also found that lower perceived control is associated with poorer cognitive performance and more rapid cognitive decline in later life (23). Individuals with lower perceived control may be less likely to initiate activities, adopt active coping and problem-solving strategies, seek health information, or engage in behaviors that may support cognitive health (24, 25). Perceived control may therefore represent an important psychological factor linking visual impairment and cognitive function and may have a potential indirect role in their association.

Social participation and perceived control may also form a serial pathway. Participation in social and community activities can provide older adults with opportunities to make choices, maintain social roles, undertake meaningful tasks, interact with others, and receive recognition and support. Such experiences may strengthen their sense of autonomy, competence, and influence over everyday life (26). When visual difficulties restrict social participation, older adults may lose opportunities to exercise choice and maintain meaningful social roles, which may be associated with lower perceived control (27). Lower perceived control may, in turn, be related to reduced cognitive engagement and poorer cognitive function (28). The stress process model offers a useful theoretical perspective for understanding this pathway (29). According to this model, chronic health problems may operate as primary stressors and give rise to secondary stressors at the social or functional level, which may subsequently be associated with psychological resources and later health outcomes. In the present study, visual impairment may be conceptualized as a persistent sensory and functional stressor, reduced social participation as a secondary social constraint, lower perceived control as an erosion of psychological resources, and poorer cognitive function as a related health outcome. Social participation and perceived control may therefore be sequentially involved in the association between visual impairment and cognitive function among older adults with diabetes, forming a potential serial pathway extending from constrained social resources to weakened psychological resources.

### The current study

Although previous research has documented associations between vision impairment and cognitive function, relatively little is known about the social and psychological pathways potentially underlying this relationship among older adults with diabetes. In particular, the independent and serial roles of social participation and perceived control have not been sufficiently examined. Understanding these pathways may provide a more comprehensive perspective on the interconnected visual, social, psychological, and cognitive challenges experienced by this population. Accordingly, this study examined the potential mediating roles of social participation and perceived control in the association between vision impairment and cognitive function among Chinese older adults with diabetes. The following hypotheses were proposed: (1) greater vision impairment would be associated with poorer cognitive function; (2) social participation and perceived control would show independent indirect associations between vision impairment and cognitive function, such that greater vision impairment would be associated with lower social participation and poorer perceived control, which would in turn be associated with lower cognitive function; and (3) social participation and perceived control would form a serial indirect pathway, whereby greater vision impairment would be associated with lower social participation, lower social participation would be associated with poorer perceived control, and poorer perceived control would subsequently be associated with lower cognitive function.

## Methods and measurements

### Data source and sample

**Figure 1:**
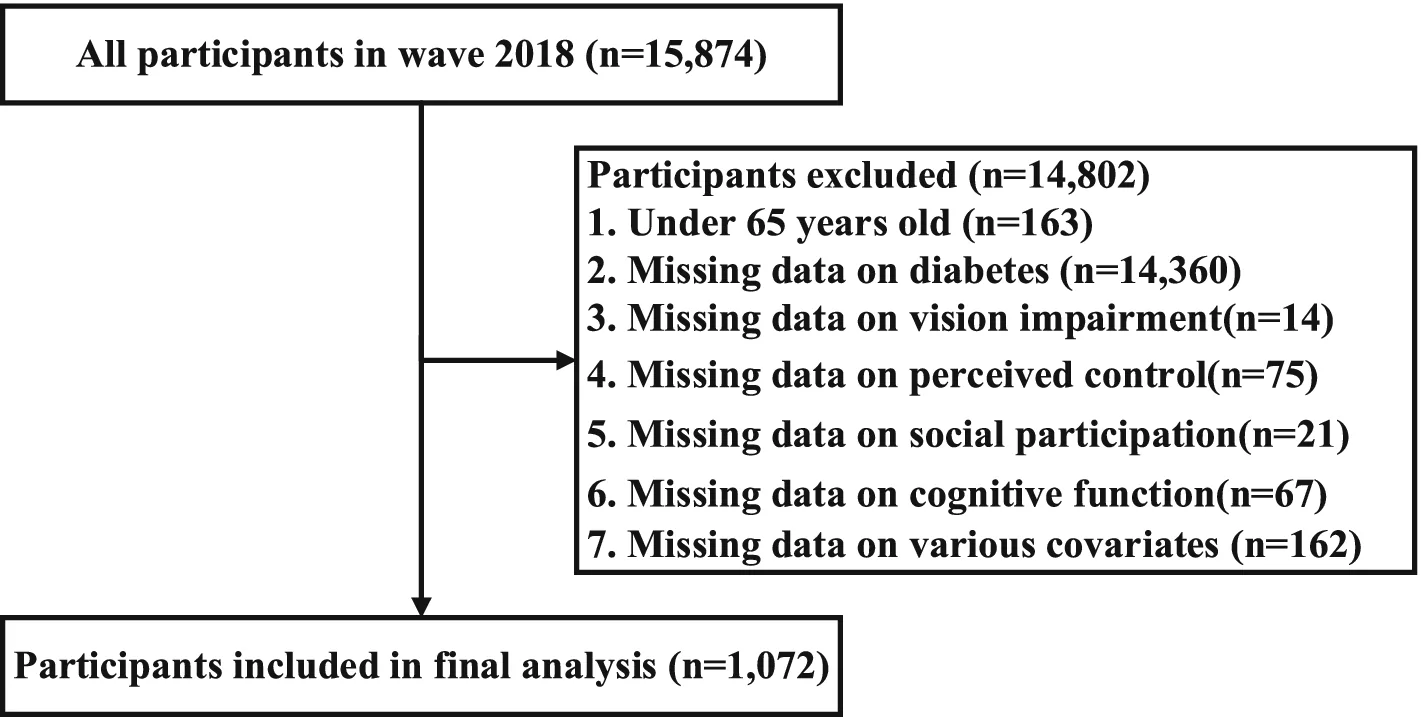
Filter flowchart.

This cross-sectional study utilized data from the 2018 wave of the Chinese Longitudinal Healthy Longevity Survey (CLHLS). The CLHLS is conducted by the Center for Healthy Aging and Development Studies at Peking University and includes adults aged 65 years and older from 23 provinces in China. Participants were recruited using a multistage, disproportionate, and targeted sampling strategy. The CLHLS database has been widely used in aging-related research in China and internationally. In the 2018 survey, health-related and sociodemographic information was collected from 15,874 older adults. The present study focused on older adults with diabetes. The main study variables included vision impairment, perceived control, social participation, and cognitive function. Sociodemographic and health-related covariates included age, sex, place of residence, living arrangement, marital status, years of education, subjective economic status, self-rated health, life satisfaction, smoking status, and alcohol consumption. Participants were excluded if they were younger than 65 years, did not have diabetes, or had missing data on the main study variables or covariates. After applying the eligibility criteria, a total of 1,072 participants with diabetes were included in the final analysis. The detailed participant selection process is presented in Figure 1.

### Measurements

#### Vision impairment

Visual impairment was assessed by asking participants whether they could see and distinguish a circle on a card under flashlight illumination without wearing glasses (30). The response options were: (1) able to see and distinguish the circle, (2) able to see but unable to distinguish it, (3) unable to see clearly, and (4) blind. Responses were scored from 1 to 4, with higher scores indicating more severe visual impairment.

#### Social participation

Social participation was measured using six items derived from the CLHLS, covering activities such as outdoor exercise, playing cards or mahjong, and involvement in organized social activities (31). Outdoor activities included Tai Chi, square dancing, visiting relatives or friends, socializing with others, and other forms of outdoor engagement. Each item was rated on a five-point frequency scale: 1 = almost every day, 2 = at least once a week, 3 = at least once a month, 4 = occasionally, and 5 = never. Higher total scores indicated lower levels of social participation.

#### Perceived control

Perceived control was assessed using the question, “Do you have the final say in matters concerning yourself?” Responses were rated on a 5-point Likert scale ranging from 1 (“always”) to 5 (“never”), with higher scores indicating lower levels of perceived control (32).

#### Cognitive function

Cognitive function was evaluated using a standardized cognitive screening scale adapted for Chinese older adults in the CLHLS (33). The instrument contains 24 items covering orientation, verbal fluency, attention, calculation, visuospatial ability, memory, and language. In the verbal fluency task, participants were asked to name as many food items as possible within 1 min, receiving 1 point for each correct response, up to a maximum of 7 points. All remaining items were scored dichotomously, with 1 point assigned for a correct answer and 0 for an incorrect answer. The total score ranges from 0 to 30, with higher scores indicating better cognitive function.

### Statistical analysis

All statistical analyses were performed using IBM SPSS Statistics version 27.0. Descriptive statistics were used to summarize the sociodemographic and health-related characteristics of the study participants. Normally distributed continuous variables were expressed as the mean ± standard deviation, whereas non-normally distributed continuous variables were presented as the median and interquartile range. Categorical variables were summarized as frequencies and percentages. Considering the measurement properties of the study variables and the objective of examining monotonic associations, Spearman’s rank correlation analysis was conducted to assess the relationships among vision impairment, social participation, perceived control, and cognitive function. Before the mediation analysis, a multiple linear regression analysis was conducted to examine the associations between potential covariates and cognitive function. Cognitive function was entered as the dependent variable, while sociodemographic and health-related characteristics were entered as independent variables. Candidate covariates included age, sex, place of residence, living arrangement, marital status, years of education, subjective economic status, self-rated health, life satisfaction, smoking status, and alcohol consumption. Variables significantly associated with cognitive function were subsequently included as covariates in the final mediation model. The serial mediating effects of social participation and perceived control on the association between vision impairment and cognitive function were examined using Model 6 of the PROCESS macro version 4.0 developed by Hayes (34). Vision impairment was entered as the independent variable, cognitive function as the dependent variable, social participation as the first mediator, and perceived control as the second mediator. The model simultaneously estimated the specific indirect effects through social participation and perceived control separately, as well as the serial indirect effect through social participation followed by perceived control. Indirect effects were estimated using a bootstrap procedure with 5,000 resamples, and bias-corrected 95% confidence intervals were calculated. An indirect effect was considered statistically significant when its 95% confidence interval did not include zero. All statistical tests were two-sided, and a *p* value < 0.05 was considered statistically significant.

### Common method biases

Harman’s single-factor test was conducted to assess the potential presence of common method bias (35). The unrotated exploratory factor analysis identified 8 factors with eigenvalues greater than 1, suggesting that the variance was not attributable to a single underlying factor. The first factor accounted for 29.36% of the total variance, which was below the commonly used threshold of 40%. These findings suggest that common method bias was unlikely to pose a serious threat to the results of the present study.

## Results

### Characteristics of samples

Table 1 presents the sociodemographic and health-related characteristics of the 1,072 older adults with diabetes. The mean age was 79.50 ± 9.49 years, and the mean duration of education was 5.42 ± 5.08 years. Of the participants, 454 (42.4%) were male and 618 (57.6%) were female. Regarding place of residence, 48.3% lived in cities, 26.7% in rural areas, and 25.0% in towns. More than half of the participants were currently married and living with their spouses (55.5%), whereas 42.0% were widowed. Only small proportions were separated (1.2%), never married (0.7%), or divorced (0.6%). Most participants lived with household members (81.3%), while 14.1% lived alone and 4.7% resided in an institution. The majority did not smoke (89.4%) or consume alcohol (90.5%). In terms of subjective economic status, most participants rated their economic condition as average (68.1%), followed by rich (20.1%), poor (7.4%), very rich (3.3%), and very poor (1.1%). Regarding self-reported quality of life, 41.0% rated their quality of life as good, 28.4% as average, and 27.0% as very good, whereas only 3.2 and 0.6% rated it as bad and very bad, respectively. With respect to self-rated health, 45.9% reported average health, 24.3% reported good health, and 7.7% reported very good health. In contrast, 20.5% rated their health as bad and 1.5% as very bad.

**Table 1:** The characteristics of the sample (*N* = 1,072).

Variables	Category	N	Mean ± SD /Percentage (%)
Age		1,072	79.50 ± 9.49
Years of schooling		1,072	5.42 ± 5.08
Sex	Male	454	42.4
	Female	618	57.6
Current residence	City	518	48.3
	Town	268	25.0
	Rural	286	26.7
Marital status	Currently Married and Living with Spouse	595	55.5
	separated	13	1.2
	Divorced	6	0.6
	Widowed	450	42.0
	Never Married	8	0.7
Co-residence of respondent	With Household Members	871	81.3
	Alone	151	14.1
	In an Institution	50	4.7
Smoking	Yes	114	10.6
	No	958	89.4
Drinking	Yes	102	9.5
	No	970	90.5
Subjective economic condition	Very rich	35	3.3
	rich	216	20.1
	So so	730	68.1
	poor	79	7.4
	Very poor	12	1.1
Self-reported quality of life	Very good	289	27.0
	Good	439	41.0
	So so	304	28.4
	Bad	34	3.2
	Very bad	6	0.6
Self-reported health	Very good	83	7.7
	Good	261	24.3
	So so	492	45.9
	Bad	220	20.5
	Very bad	16	1.5

SD, Standard Deviation.

Taking cognitive function as the dependent variable, a multiple linear regression analysis was conducted to examine its associations with the potential covariates. The results showed that age, years of education, sex, place of residence, self-reported quality of life, and self-rated health were significantly associated with cognitive function among older adults with diabetes (*p* < 0.05). (Table 2).

**Table 2:** Multiple linear regression analysis of factors associated with cognitive function among older adults with diabetes (*N* = 1,072).

Variables	B	SE	t	*p*
Age	−0.220	0.017	−12.910	**<0.001**
Years of schooling	0.121	0.033	3.642	**<0.001**
Sex	−0.745	0.321	−2.326	**0.020**
Current residence	−0.371	0.184	−2.021	**0.044**
Marital status	−0.070	0.118	−0.595	0.552
Co-residence of the respondent	0.112	0.291	0.384	0.701
Smoking	0.279	0.484	0.577	0.564
Drinking	0.076	0.499	0.152	0.880
Subjective economic condition	−0.303	0.228	−1.325	0.185
Self-reported quality of life	−0.704	0.187	−3.759	**<0.001**
Self-reported health	−0.447	0.176	−2.544	**0.011**

Bold text indicates *p* < 0.05.

### Correlations among vision impairment, perceived control, social participation, and cognitive function in older adults with diabetes

Table 3 presents the descriptive statistics and correlations among the study variables. Vision impairment was significantly and positively correlated with poorer perceived control (*r =* 0.136) and lower levels of social participation (*r* = 0.184), and negatively correlated with cognitive function (*r* = −0.240). Poorer perceived control was also positively correlated with lower social participation (*r* = 0.156) and negatively correlated with cognitive function (*r* = −0.195). In addition, lower social participation was significantly associated with poorer cognitive function (*r* = −0.287). All correlations were statistically significant (*p* < 0.01).

**Table 3:** Correlations among vision impairment, perceived control, social participation, and cognitive function in older adults with diabetes.

Variables	Mean ± SD	Vision impairment	Perceived control	Social participation	Cognitive function
vision impairment	1.40 ± 0.71	1			
perceived control	2.08 ± 1.11	0.136^*^	1		
social participation	25.10 ± 4.30	0.184^*^	0.156^*^	1	
cognitive function	26.49 ± 5.12	−0.240^*^	−0.195^*^	−0.287^*^	1

All values in the table represent Spearman’s rank correlation coefficients (r); *p < 0.01 (two-tailed).

### Mediation analysis of social participation and perceived control

A bootstrap-based serial multiple mediation analysis was conducted to examine the mediating roles of social participation and perceived control in the association between vision impairment and cognitive function. Age, years of schooling, sex, place of residence, self-reported quality of life, and self-rated health were included as covariates in the mediation model (Table 4). Greater vision impairment was significantly associated with poorer social participation (B = 0.4295, *p* < 0.05), whereas its association with perceived control was not statistically significant after social participation and the covariates were taken into account (B = 0.0713, *p* > 0.05). Poorer social participation was significantly associated with poorer perceived control (B = 0.0244, *p* < 0.01). After adjustment for the covariates and the other variables in the model, poorer social participation (B = −0.1128, *p* < 0.001) and poorer perceived control (B = −0.3651, *p* < 0.01) were both significantly associated with lower cognitive function. The direct association between vision impairment and cognitive function remained statistically significant after social participation and perceived control were included in the model (B = −0.8085, *p* < 0.001).

**Table 4:** A serial multiple mediation model of the association between vision impairment and cognitive function among older adults with diabetes.

Predictors	Social participation			Perceived control			Cognitive function		
	B	SE	t	B	SE	t	B	SE	t
Age	0.1168	0.0137	8.5349^***^	0.0067	0.0038	1.7544	−0.1897	0.0156	−12.1937^***^
Years of schooling	−0.1178	0.0295	−3.9987^***^	−0.0215	0.0080	−2.6805^**^	0.0913	0.0327	2.7924^**^
Sex	−0.3884	0.2648	−1.4670	0.0582	0.0717	0.8107	−0.7077	0.2912	−2.4304^*^
Current residence	0.5107	0.1631	3.1310^**^	0.0283	0.0443	0.6383	−0.3120	0.1800	−1.7335
Self-reported quality of life	0.1749	0.1628	1.0743	0.1140	0.0441	2.5861^**^	−0.6352	0.1795	−3.5387^***^
Self-reported health	0.5938	0.1553	3.8240^***^	0.1155	0.0423	2.7293^**^	−0.3111	0.1723	−1.8054
Vision Impairment	0.4295	0.1788	2.4026^*^	0.0713	0.0485	1.4704	−0.8085	0.1971	−4.1028^***^
Social Participation				0.0244	0.0083	2.9394^**^	−0.1128	0.0338	−3.3367^***^
Perceived Control							−0.3651	0.1245	−2.9338^**^
R^2^	0.1535			0.0731			0.2791		
F	27.5541			10.4796			45.6913		

B, unstandardized regression coefficient, SE, Standard Error, *p < 0.05, **p < 0.01, ***p < 0.001.

**Figure 2:**
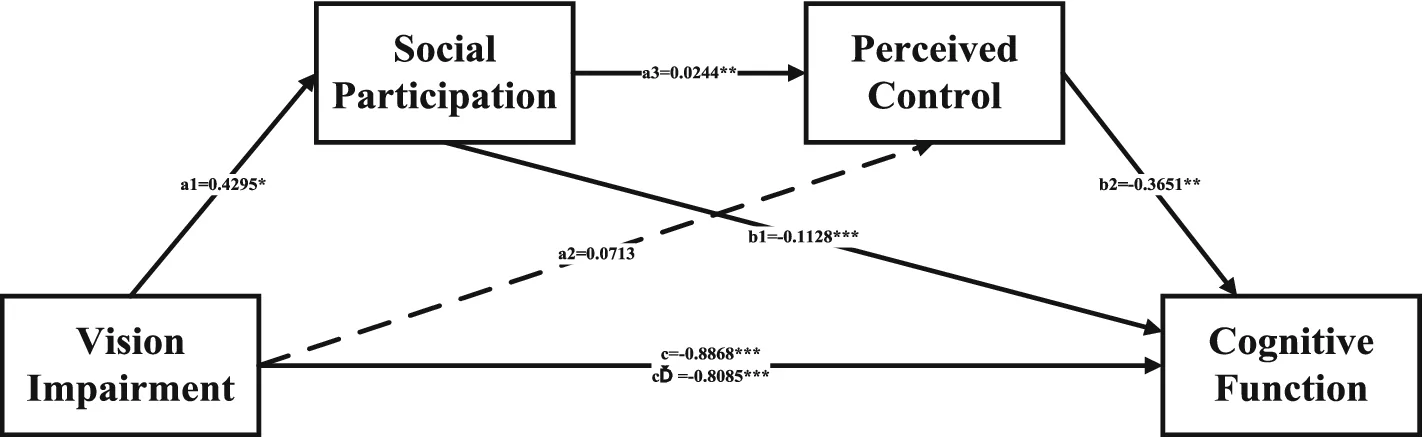
Serial mediation model of vision impairment, social participation, perceived control, and cognitive function. Note: The path coefficients have been shown in the standardized regression coefficients. *p < 0.05, **p < 0.01, ***p < 0.001.

The results of the serial mediation analysis examining the potential mediating roles of social participation and perceived control in the association between vision impairment and cognitive function are presented in Figure 2 and Table 5. The total association between vision impairment and cognitive function was statistically significant, indicating that greater vision impairment was associated with poorer cognitive function among older adults with diabetes.

**Table 5:** Total, direct, and indirect effects of vision impairment on cognitive function through social participation and perceived control among older adults with Diabetes.

Pathway	Effect	SE	BootLLCI	BootULCI
Total effect (c)	−0.8868	0.1981	−1.2756	−0.4981
Direct effect (c’)	−0.8085	0.1971	−1.1952	−0.4218
a1	0.4295	0.1788	0.0787	0.7803
a2	0.0713	0.0485	**−0.0239**	**0.1665**
a3	0.0244	0.0083	0.0081	0.0407
b1	−0.1128	0.0338	−0.1791	−0.0465
b2	−0.3651	0.1245	−0.6094	−0.1209
Indirect effects				
Total indirect effects	−0.0783	0.0317	−0.1466	−0.0215
Indirect 1	−0.0484	0.0226	−0.0965	−0.0089
Indirect 2	−0.0260	0.0235	**−0.0830**	**0.0090**
Indirect 3	−0.0038	0.0026	−0.0102	−0.0002

Indirect effect 1, vision impairment → social participation → cognitive function; indirect effect 2, vision impairment → perceived control → cognitive function; indirect effect 3, vision impairment → social participation → perceived control → cognitive function. BootLLCI self-help lower confidence interval, BootULCI self-help upper confidence interval, SE standard error, effect standardized regression coefficient. The bolded part indicates that the confidence interval includes 0.

In addition, the specific indirect pathways were examined. The first indirect pathway, vision impairment → social participation → cognitive function, was statistically significant, with an indirect effect of −0.0484 (Boot 95% CI: −0.0965 to −0.0089). This finding indicates that greater vision impairment was associated with lower social participation, which was subsequently associated with poorer cognitive function. The second indirect pathway, vision impairment → perceived control → cognitive function, was not statistically significant because its bootstrap confidence interval included zero (indirect effect = −0.0260, Boot 95% CI: −0.0830 to 0.0090). The third indirect pathway, vision impairment → social participation → perceived control → cognitive function, was statistically significant, with an indirect effect of −0.0038 (Boot 95% CI: −0.0102 to −0.0002). Social participation was also significantly associated with perceived control (B = 0.0244, *p* < 0.01), suggesting that lower social participation was associated with poorer perceived control. The total indirect effect was −0.0783 (Boot 95% CI: −0.1466 to −0.0215), accounting for approximately 8.83% of the total effect. Specifically, the indirect pathway through social participation accounted for approximately 5.46% of the total effect, the pathway through perceived control accounted for approximately 2.93%, and the serial pathway through social participation and perceived control accounted for approximately 0.43%. However, because the indirect effect through perceived control alone was not statistically significant, its proportion should be interpreted with caution. The direct effect of vision impairment on cognitive function remained statistically significant after the mediators were included (effect = −0.8085, 95% CI: −1.1952 to −0.4218), indicating a partial mediation pattern. Taken together, these findings suggest that social participation may independently mediate the association between vision impairment and cognitive function and may also operate sequentially through perceived control. In contrast, perceived control alone did not significantly mediate this association.

Sensitivity analyses using different covariate-adjustment strategies yielded results generally consistent with those of the primary model. The direct effect, total indirect effect, indirect effect through social participation alone, and serial indirect effect through social participation and perceived control remained statistically significant in both the unadjusted and fully adjusted models. The indirect effect through perceived control alone was significant in the unadjusted model but became non-significant after covariate adjustment. Notably, the effect estimates and significance patterns in the fully adjusted model were highly consistent with those of the primary model (Supplementary Tables S1, S2).

## Discussion

Based on the proposed hypotheses and the findings of this study, we explored the potential mechanisms underlying the association between vision impairment and cognitive function among older adults with diabetes. Specifically, we examined whether social participation and perceived control might represent potential mediating pathways in this association. Greater vision impairment was associated with poorer cognitive function. Social participation showed a significant indirect association between vision impairment and cognitive function, whereas the indirect pathway through perceived control alone was not statistically significant. In addition, a significant serial indirect pathway was identified, in which greater vision impairment was associated with lower social participation, lower social participation was associated with poorer perceived control, and poorer perceived control was further associated with lower cognitive function. These findings suggest that social participation and perceived control may be involved in the association between vision impairment and cognitive function.

### The direct association between vision impairment and cognitive function

The present study found that vision impairment was significantly associated with poorer cognitive function among older adults with diabetes, which is broadly consistent with previous research (8, 36). This finding suggests that visual difficulties may be an important correlate of cognitive health in this population. Vision impairment may be associated with reduced visual sensory input, greater cognitive demands when acquiring and processing environmental information, and fewer opportunities to engage in socially and cognitively stimulating activities, all of which may be related to poorer cognitive performance (9, 37). In addition, according to the cognitive compensation hypothesis, impaired visual input may require individuals to allocate more of their limited cognitive resources to visually demanding tasks, leaving fewer resources available for attention, memory encoding, and other higher-order cognitive processes. Such competition for cognitive resources may be particularly disadvantageous for older adults with lower cognitive reserve (38, 39). Among older adults with diabetes, the association between vision impairment and cognitive function may be particularly complex. Persistent hyperglycemia, endothelial dysfunction, and microvascular damage associated with diabetes may affect both the retinal and cerebral microvasculature. Thus, the observed association between vision impairment and poorer cognitive function may partly reflect shared metabolic and neurovascular pathological processes (36, 40). Meanwhile, multimorbidity and functional limitations, which are common in later life, together with limited access to ophthalmic examinations and vision rehabilitation services, may further contribute to the co-occurrence of visual and cognitive problems (41, 42). The possibility of reciprocal or complex relationships should also be considered. Although vision impairment may be associated with poorer cognitive function, cognitive decline may also reduce individuals’ ability to recognize visual problems and adhere to diabetes- and eye-care recommendations. Conversely, vision impairment itself may affect performance on cognitive test items that rely on visual stimuli (43, 44). Therefore, the association observed in the present study should not be interpreted as evidence that vision impairment causes cognitive decline. Future longitudinal studies incorporating objective ophthalmological examinations and repeated cognitive assessments are needed to clarify the temporal sequence and direction of these relationships.

### The mediating effects of social participation and perceived control

This study employed a serial multiple mediation model to further examine the potential roles of social participation and perceived control in the association between visual impairment and cognitive function among older adults with diabetes. The results showed that more severe visual impairment was significantly associated with lower levels of social participation, which, in turn, were significantly associated with poorer cognitive function. The indirect association between visual impairment and cognitive function through social participation was statistically significant and accounted for approximately 5.46% of the total association. This finding supported the hypothesized independent indirect pathway through social participation and suggested that reduced social participation may represent an important and potentially modifiable correlate when considering the relationship between visual difficulties and cognitive health among older adults with diabetes.

Visual impairment may be associated with reduced social participation through several interrelated mechanisms. A diminished ability to see and identify objects may restrict independent mobility, reduce confidence in navigating unfamiliar environments, and heighten concerns about falls or other accidents during outdoor activities (42, 45). Visual limitations may also make it more difficult to participate in activities that rely heavily on visual information, such as reading, playing cards or mahjong, visiting relatives and friends, and attending organized community activities (13, 46). Consequently, older adults with visual impairment may have fewer opportunities for social interaction, physical activity, and cognitive stimulation. Previous studies have suggested that social participation may be related to cognitive health by providing opportunities for interpersonal communication, emotional support, cognitive stimulation, and the maintenance of meaningful social roles (17, 47). Accordingly, the significant indirect pathway identified in this study may reflect a pattern in which visual difficulties are associated with reduced engagement in social activities and cognitively stimulating environments, which is, in turn, associated with poorer cognitive function. However, given the cross-sectional design of this study, the possibility that poorer cognitive function may also limit social participation cannot be excluded.

In another pathway, lower perceived control was significantly associated with poorer cognitive function. However, after accounting for social participation and relevant covariates, the direct association between visual impairment and perceived control was not statistically significant. Perceived control therefore did not show a significant independent indirect association between visual impairment and cognitive function. This finding was not fully consistent with the original hypothesis and suggested that visual impairment alone may be insufficient to explain differences in perceived control among older adults with diabetes. Perceived control may also be shaped by multiple factors, including functional independence, family support, financial resources, the burden of disease management, access to healthcare services, and the ability to make decisions regarding everyday affairs (20, 48, 49). Some older adults with visual difficulties may retain a relatively strong sense of control when adequate assistance, environmental adaptations, or compensatory strategies are available (15). Although the independent indirect pathway through perceived control was not statistically significant, perceived control remained significantly associated with cognitive function. A lower sense of control may be associated with less frequent use of adaptive or compensatory strategies and with lower levels of social, cognitive, and physical activity, which may, in turn, be related to poorer cognitive health (50, 51). From a broader disability and mental health perspective, the psychological consequences of visual and participation limitations may extend beyond perceived control. Restricted opportunities for independent activity, social role participation, and meaningful engagement may also be associated with reduced autonomy, poorer subjective well-being, and worse emotional health. Previous studies linking vision impairment and reduced social participation with lower well-being or greater depressive symptoms provide convergent support for this broader psychosocial context (52, 53). Perceived control may therefore represent only one component of a wider range of psychological resources potentially affected by visual and participation limitations, which may partly explain its relatively weak independent indirect association in the present study. At the same time, poorer cognitive function may itself reduce confidence in one’s decision-making ability and weaken the perceived capacity to manage everyday affairs (54). The association between perceived control and cognitive function observed in this study may therefore be bidirectional rather than reflecting a unidirectional process.

Notably, social participation and perceived control jointly formed a statistically significant serial indirect pathway, although this pathway accounted for only approximately 0.43% of the total association between visual impairment and cognitive function. Specifically, more severe visual impairment was associated with lower social participation; lower social participation was, in turn, associated with lower perceived control; and lower perceived control was further associated with poorer cognitive function. Although statistically significant, the magnitude of this serial indirect effect was very small. Therefore, it should be interpreted as evidence of a possible psychosocial pathway rather than as a major mechanism underlying the association between vision impairment and cognitive function. Its statistical significance should not be taken to imply substantial practical or clinical importance. Participation in social and community activities may provide older adults with opportunities to make choices, maintain social roles, complete meaningful tasks, and receive feedback and recognition from others (55). When such opportunities are restricted, individuals may experience a reduced sense of autonomy, competence, and influence over everyday life (56). Lower perceived control may also be associated with reduced cognitive engagement and less active coping with challenges related to aging or chronic illness (50). These observations provide a plausible context for the serial association identified in this study, but the small effect size and cross-sectional design warrant caution in assigning this pathway a prominent explanatory role.

Within the Chinese sociocultural context, the pathways identified in this study may have particular practical relevance. Social participation among older Chinese adults commonly includes visiting relatives and friends, playing cards or mahjong, and participating in activities such as square dancing or tai chi. Many of these activities require adequate vision, independent mobility, and confidence in navigating public or community spaces. Older adults with visual impairment may gradually reduce their participation in such activities because of safety concerns, embarrassment, transportation difficulties, or insufficiently accessible environments (57, 58). These challenges may be more pronounced among older adults with diabetes, who may simultaneously experience physical functional limitations, treatment burden, and concerns about diabetes-related complications (59, 60). In addition, some older adults may regard declining vision as a normal part of aging rather than as a health problem requiring professional assessment and treatment (61). Delayed eye examinations and insufficient correction of visual problems may therefore be associated with prolonged limitations in daily activities and social participation (62). Urban–rural differences in access to ophthalmic care, vision rehabilitation resources, and community activity programs may also shape how older adults respond to visual impairment (63). Family support may help compensate for some of the difficulties associated with visual limitations by assisting with mobility, medication management, and daily activities (42). However, when family members take over tasks that older adults may still be capable of performing, they may unintentionally reduce opportunities for independent decision-making and autonomous participation, which may be associated with lower perceived control (64). Supportive measures should therefore seek to balance safety and necessary assistance with the preservation of autonomy, social participation, and meaningful choice.

In summary, the present mediation model provides a more detailed understanding of the potential social and psychological pathways underlying the association between vision impairment and cognitive function among older adults with diabetes. Social participation showed a statistically significant independent indirect association, whereas the indirect pathway through perceived control alone did not reach statistical significance. Nevertheless, social participation and perceived control jointly formed a significant serial indirect pathway. However, because the serial indirect effect accounted for only a very small proportion of the total association, its statistical significance should be distinguished from its practical importance. The findings therefore provide preliminary evidence for a possible pathway involving social participation and perceived control rather than demonstrating that this pathway constitutes a major mechanism or intervention target. These findings highlight the potential importance of maintaining social engagement among older adults with visual difficulties and suggest that perceived control may be particularly relevant when considered within the context of restricted social participation. Because of the cross-sectional design, longitudinal studies are needed to determine whether changes in vision, social participation, and perceived control precede subsequent changes in cognitive function.

### Practical implications

To date, research examining the potential functional and psychosocial mechanisms underlying the association between vision impairment and cognitive function among older adults with diabetes remains limited, particularly regarding the roles of social participation and perceived control. The findings of this study may have implications for diabetes management, geriatric care, community health services, and public health practice. Given the significant indirect pathway through social participation and the serial pathway involving social participation and perceived control, assessments of older adults with diabetes may benefit from considering visual functioning, opportunities for social engagement, perceived control over daily life, and cognitive function together rather than evaluating these factors in isolation. Routine follow-up in primary care and diabetes management settings could incorporate brief screening for difficulties in seeing and recognizing objects, reduced participation in outdoor or organized activities, diminished autonomy in everyday decision-making, and possible cognitive difficulties. Older adults reporting vision problems could be referred for timely ophthalmic assessment and appropriate management, including correction of refractive errors, treatment of eye diseases, provision of visual aids, and guidance on environmental adaptations. At the same time, community-based services could provide accessible opportunities for social interaction and participation, such as group exercise, recreational activities, peer-support programs, and home- or neighborhood-based activities for individuals with mobility or visual limitations. Although perceived control alone did not show a statistically significant indirect effect, it was involved in the significant serial indirect pathway following social participation. This finding suggests that perceived control may be particularly relevant in the context of restricted social engagement. Health professionals and caregivers could therefore support older adults in maintaining involvement in daily decisions, selecting preferred activities, and managing achievable aspects of their health and daily routines. Greater collaboration among diabetes care, ophthalmology, geriatric medicine, rehabilitation, cognitive health, and community social services may help address the interconnected visual, functional, psychosocial, and cognitive needs of this population. However, because the present study was cross-sectional, these implications should be interpreted cautiously. Longitudinal and intervention studies are needed to clarify the temporal relationships among vision impairment, social participation, perceived control, and cognitive function and to determine whether changes in social participation or perceived control are associated with subsequent cognitive outcomes.

### Limitations

Several limitations of this study should be acknowledged. First, the cross-sectional design precluded determination of the temporal sequence among vision impairment, social participation, perceived control, and cognitive function. Therefore, the observed indirect associations should not be interpreted as evidence of causality, and longitudinal studies are needed to clarify the direction and temporal ordering of these relationships. Second, diabetes status was identified from survey information and was not verified using medical records, fasting plasma glucose, or glycated hemoglobin measurements. In addition, detailed information on diabetes duration, glycemic control, treatment, and diabetes-related complications was not available. Future research should incorporate more comprehensive metabolic and ophthalmological assessments when such data are available. Third, complete-case analysis was used to handle missing data for the primary variables and covariates. This approach may have introduced selection bias if participants with incomplete data differed systematically from those included in the analysis. Consequently, the findings may not be fully generalizable to all older adults with diabetes. Future studies could apply multiple imputation or other appropriate missing-data methods and compare the characteristics of included and excluded participants. Fourth, although the CLHLS covers multiple provinces and includes a large and diverse sample of Chinese older adults, its sampling strategy oversamples the oldest-old population. Sampling weights were not applied in the present study; therefore, the descriptive findings should not be interpreted as nationally representative prevalence estimates or population distributions for all older adults with diabetes in China. The external generalizability of the findings should therefore be interpreted with caution. Finally, although the mediation model adjusted for age, years of education, sex, place of residence, self-reported quality of life, and self-rated health, other potentially important confounders were not fully considered. Future studies should incorporate a broader range of clinical, psychological, behavioral, and environmental factors to further assess the robustness of the observed associations.

## Conclusion

Despite these limitations, this study provides meaningful insights into the associations among vision impairment, social participation, perceived control, and cognitive function in older adults with diabetes. Greater vision impairment was associated with poorer cognitive function, and social participation showed a significant indirect association in this relationship. A significant serial indirect pathway involving social participation and perceived control was also observed, whereas the indirect pathway through perceived control alone was not statistically significant. These findings suggest that social participation may represent an important functional and psychosocial factor in the association between vision impairment and cognitive function, while perceived control may be particularly relevant when considered together with social participation. The results highlight the value of jointly considering visual function, social engagement, perceived control, and cognitive health in the assessment and support of older adults with diabetes. Given the cross-sectional design, longitudinal studies are needed to clarify the temporal sequence and direction of these associations.

## Data Availability

The datasets presented in this study can be found in online repositories. The names of the repository/repositories and accession number(s) can be found at: the data used in this study are publicly available. The data can be obtained at: https://opendata.pku.edu.cn/dataverse/CHADS;jsessionid=ebc0450d9506942ccd901fc1a322. Researchers are required to apply for permission to use the data.
