# Supplementary material for: Vision impairment and cognitive function in Chinese older adults with diabetes: multiple mediating effects of social participation and perceived control
Source: Front Public Health. 2026 Sep 9;14:1952426. doi: 10.3389/fpubh.2026.1952426 (PMC13597311; doi:10.3389/fpubh.2026.1952426)
Supplement: Supplementary file 1 [file Table_1.DOCX]

Supplementary Table S1. Unadjusted direct and indirect effects of vision impairment on cognitive function through social participation and perceived control

| **Pathway** | **Effect** | **SE** | **BootLLCI** | **BootULCI** |
| --- | --- | --- | --- | --- |
| Total effect (c) | -1.8840 | 0.2116 | -2.2992 | -1.4687 |
| Direct effect (c’) | -1.5010 | 0.2075 | -1.9082 | -1.0937 |
| a1 | 1.0550 | 0.1816 | 0.6986 | 1.4114 |
| a2 | 0.1400 | 0.0476 | 0.0466 | 0.2334 |
| a3 | 0.0389 | 0.0079 | 0.0234 | 0.0544 |
| b1 | -0.2590 | 0.0346 | -0.3269 | -0.1910 |
| b2 | -0.6065 | 0.1328 | -0.8671 | -0.3458 |
| Indirect effects |  |  |  |  |
| Total indirect effects | -0.3830 | 0.0660 | -0.5170 | -0.2597 |
| Indirect 1 | -0.2732 | 0.0534 | -0.3831 | -0.1742 |
| Indirect 2 | -0.0849 | 0.0391 | -0.1747 | **-0.0211** |
| Indirect 3 | -0.0249 | 0.0091 | -0.0458 | -0.0104 |

Supplementary Table S2. Fully adjusted direct and indirect effects of vision impairment on cognitive function through social participation and perceived control

| **Pathway** | **Effect** | **SE** | **BootLLCI** | **BootULCI** |
| --- | --- | --- | --- | --- |
| Total effect (c) | -0.8796 | 0.1986 | -1.2692 | -0.4899 |
| Direct effect (c’) | -0.8035 | 0.1974 | -1.1909 | -0.4161 |
| a1 | 0.4050 | 0.1780 | 0.0557 | 0.7542 |
| a2 | 0.0718 | 0.0483 | **-0.0229** | **0.1665** |
| a3 | 0.0270 | 0.0083 | 0.0106 | 0.0433 |
| b1 | -0.1094 | 0.0341 | -0.1764 | -0.0424 |
| b2 | -0.3642 | 0.1256 | -0.6306 | -0.1377 |
| Indirect effects |  |  |  |  |
| Total indirect effects | -0.0761 | 0.0323 | -0.1479 | -0.0202 |
| Indirect 1 | -0.0443 | 0.0213 | -0.0898 | -0.0072 |
| Indirect 2 | -0.0276 | 0.0242 | **-0.0863** | **0.0098** |
| Indirect 3 | -0.0042 | 0.0029 | -0.0113 | -0.0003 |
